# Enolase and dipeptidyl peptidase IV protein sub-unit vaccines are not protective against a lethal *Streptococcus suis* serotype 2 challenge in a mouse model of infection

**DOI:** 10.1186/s12917-019-2196-y

**Published:** 2019-12-10

**Authors:** Audrey Dumesnil, Léa Martelet, Daniel Grenier, Jean-Philippe Auger, Josée Harel, Eric Nadeau, Marcelo Gottschalk

**Affiliations:** 1 Groupe de recherche sur les maladies infectieuses en production animale (GREMIP), Faculty of Veterinary Medicine, University of Montreal, 3200 Sicotte St.,, Saint-Hyacinthe, QC J2S 2M2 Canada; 2 Swine and Poultry Infectious Diseases Research Center (CRIPA), Montreal, Quebec, Canada; 3 Oral Ecology Research Group (GREB), Faculty of Dentistry, Laval University, Quebec City, Quebec, Canada; 4 Prevtec Microbia Inc. 3395 Casavant W. Blvd, Saint-Hyacinthe, QC J2S 0B8 Canada

**Keywords:** *Streptococcus suis* serotype 2, Sub-unit vaccines, Antibody production, Protection, Mouse model, Experimental design, bias

## Abstract

**Background:**

*Streptococcus suis* is a major swine pathogen causing arthritis, meningitis and sudden death in post-weaning piglets and is also a zoonotic agent. *S. suis* comprises 35 different serotypes of which the serotype 2 is the most prevalent in both pigs and humans. In the absence of commercial vaccines, bacterins (mostly autogenous), are used in the field, with controversial results. In the past years, the focus has turned towards the development of sub-unit vaccine candidates. However, published results are sometimes contradictory regarding the protective effect of a same candidate. Moreover, the adjuvant used may significantly influence the protective capacity of a given antigen. This study focused on two protective candidates, the dipeptidyl peptidase IV (DPPIV) and the enolase (SsEno). Both proteins are involved in *S. suis* pathogenesis, and while contradictory protection results have been obtained with SsEno in the past, no data on the protective capacity of DPPIV was available.

**Results:**

Results showed that among all the field strains tested, 86 and 88% were positive for the expression of the SsEno and DPPIV proteins, respectively, suggesting that they are widely expressed by strains of different serotypes. However, no protection was obtained after two vaccine doses in a CD-1 mouse model of infection, regardless of the use of four different adjuvants. Even though no protection was obtained, significant amounts of antibodies were produced against both antigens, and this regardless of the adjuvant used.

**Conclusions:**

Taken together, these results demonstrate that *S. suis* DPPIV and SsEno are probably not good vaccine candidates, at least not in the conditions evaluated in this study. Further studies in the natural host (pig) should still be carried out. Moreover, this work highlights the importance of confirming results obtained by different research groups.

## Background

*Streptococcus suis* (*S. suis*) is one of the most frequent causes of mortality in weaned piglets worldwide, causing mainly septicemia with sudden death, meningitis and arthritis (1). It is also considered an emerging zoonotic agent, mainly in South-East Asia, as an etiological agent of meningitis and septic shock (2, 3). *S. suis* is classified into 35 serotypes based on the antigenicity of the capsular polysaccharide (CPS). More recently, serotypes 20, 22, 26, 32, 33, and 34 have been suggested to belong to different bacterial species (4), whereas strains with new CPS genes have also been described (5). Serotype 2 is reported as being the most virulent and frequently recovered serotype from diseased animals (6). However, other serotypes have also been described to be able to cause serious diseases, mainly 5, 7, 9 and 14 (7). In humans, serotype 2 is also by far the serotype most frequently recovered from ill patients, followed by serotype 14 (6).

Early steps of the *S. suis* infection mainly take place in the upper respiratory and, as more recently suggested, the intestinal tract, where bacteria adhere to and, to a certain extent, invade epithelial cells (8). Although the mechanisms are not completely understood, *S. suis* eventually reaches the bloodstream, remains extracellular by resisting phagocytosis, and causes disease (9). *S. suis* resistance to phagocytosis by professional phagocytes is mainly due to the presence of the CPS (9). It is not unusual to have more than one serotype (and sometimes, different strains of the same serotype) involved in clinical cases in a given herd (3).

Early medicated weaning and segregated early weaning practices do not eliminate *S. suis* infection (3). Therefore, effective control measures to prevent disease depend on control of predisposing factors, prophylactic/metaphylactic procedures (where allowed) and/or vaccination (3). Field reports describing vaccine failure are common (10, 11). Indeed, commercial vaccines are almost inexistent and those used in the field are mostly autogenous bacterins (10). With some exceptions, a limited protective response is usually reported with bacterins, which may be attributed to failure of the whole-bacterial antigens to elicit an immune response. This defective immunogenicity may be due, at least in part, to the presence of a low immunogenic CPS, to the loss of antigenicity caused by heat or formalin processing, production of antibodies to antigens not associated with protection, serotype-specific protection (when different serotypes are inducing disease in a given herd), and/or other unknown reasons (10).

Most research studies on *S. suis* vaccines have been performed with sub-unit candidates, which are based on proteins, with the exception of a serotype-specific CPS-conjugate vaccine (12). The main objective of protein-based sub-unit vaccines is usually to obtain a highly immunogenic cross-reactive antigen that would eventually protect against different serotypes (and strains) of *S. suis*. Indeed, more than 40 protein candidates have been shown to induce either protective antibodies (through in vitro opsonophagocytosis tests) or protection after in vivo challenge, mostly with serotype 2 strains (10, 13–16). With the exception of the suilysin and Sao proteins, which have been reported to be relatively constant in protection (with a few exceptions) when tested by independent research groups (10, 17), other vaccine candidates either presented contradictory results or were never tested by independent research groups (10). To further complicate the interpretation of results, it has been demonstrated that a given protein may either be protective or non-protective depending on the adjuvant used (10, 18, 19).

In the present study, two sub-unit vaccine candidates, the dipeptidyl peptidase IV (DPPIV) and the enolase (SsEno), which are both membrane-associated proteins, were tested in a mouse model of infection in combination with one of four different adjuvants. The DPPIV, although not critical for virulence, has been described to play important functions in the pathogenesis of the infection caused by *S. suis* (20), but has never been tested as a vaccine candidate. The SsEno is a protein that has been described as playing important roles as a virulence factor (21, 22), but presented contradictory results when used in vaccination trials (23, 24). The presence of such vaccine protein candidates in a large collection of field strains of *S. suis* belonging to different serotypes has also never been tested.

## Results

### Production of DPPIV and SsEno by field strains of *S. suis* belonging to different serotypes

**Fig. 1:**
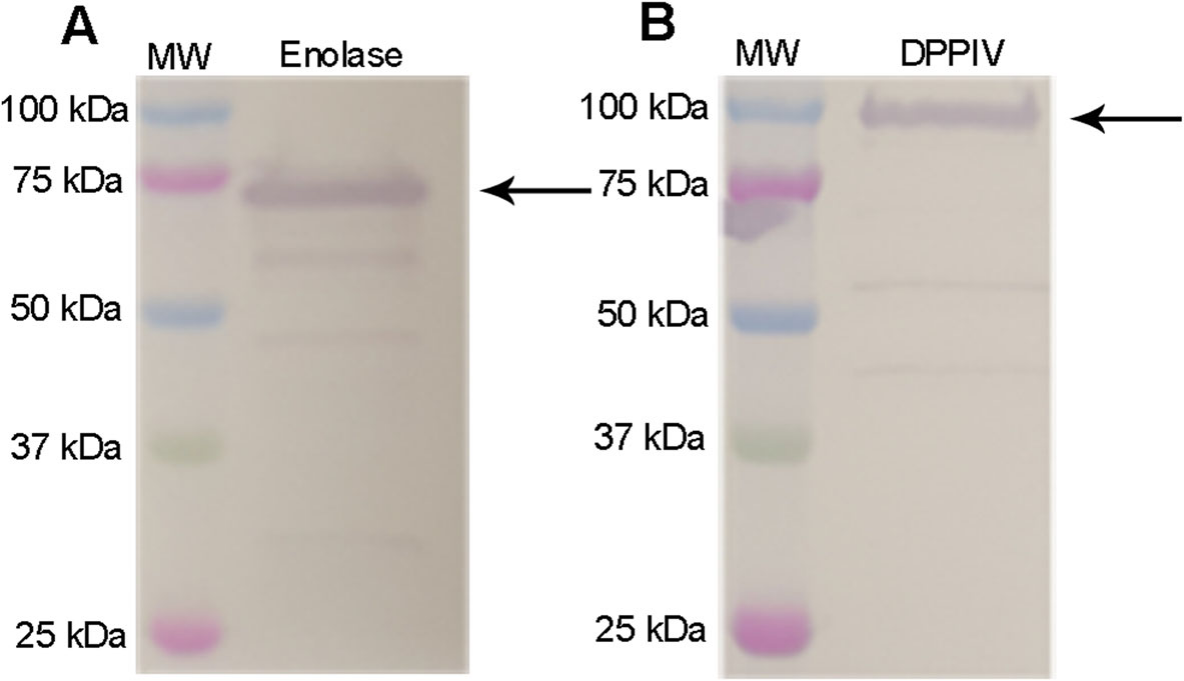
Recognition of enolase (A) and DPPIV (B) with specific antisera produced against theses antigens. The black arrows correspond to the purified protein and MW to the molecular weight ladder (lane 1)

Antisera produced against recombinant enolase (rSsEno**)** and DPPIV (rDPPIV) showed clear reactions with purified proteins of expected molecular masses of approximately 75 kDa and 100 kDa, respectively (Fig. 1A and B). The constitutional expression of SsEno and DPPIV was then evaluated in *S. suis* field strains by dot-blot with these mono-specific polyclonal hyperimmune rabbit sera. At least one of the two proteins were found in all *S. suis* serotypes, with the exception of the only serotype 6 strain available (Table 2). In the case of SsEno, 86% of all field strains belonging to all other serotypes were recognized by the anti sera against the rSsEno. Moreover, the DPPIV protein was expressed by 88% of the field strains tested belonging to all serotypes, with the exception of serotype 6 (as mentioned above), and the only available serotype 32 strain.

### Protection assays

**Fig. 2:**
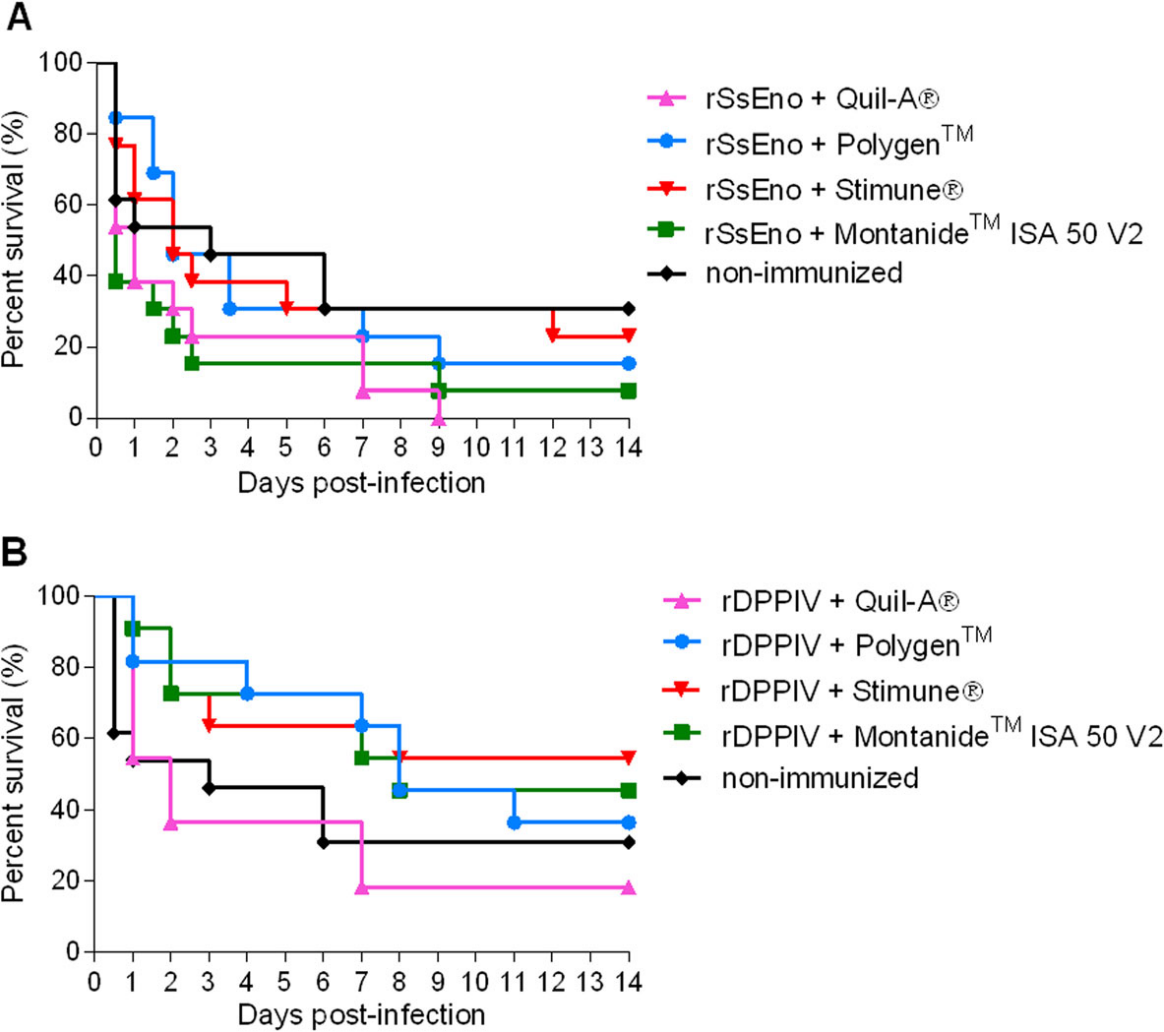
The combination of rSsEno or rDPPIV with 4 different adjuvants is not protective against *S. suis*. Survival of the mice immunized with 50 μg of rSsEno (A) or rDPPIV (B) with either Polygen™, Montanide™ ISA 50 V2, Quil-A® or Stimune® adjuvants following challenge with *S. suis* 31533 strain. A control group (non-immunized) received 100 μL of phosphate-buffered saline

Twenty-four hours after challenge infection with the 31533 strain of *S. suis* serotype 2, mice from all groups began to present severe clinical signs of disease including rough hair coat, depression, prostration, and general weakness, leading to the euthanasia of certain individuals. Mortality during the first 5 days was mostly due to septicemia with bacterial colonization of systemic organs (data not shown). However, from day 6 post-challenge, several of the mice developed severe signs of central nervous system infection including pedaling, running in circles and opisthotonos. At least 8/11 mice immunized with rSsEno (independently of the adjuvant used) died or were euthanatized for ethical reasons at the end of the experiment (14 days post-infection) (Fig. 2A). In addition, less than 7/13 animals immunized with the rDPPIV and the different adjuvants survived 14 days post-infection (Fig. 2B). Indeed, at the end of the trial, no significant differences in survival between the control group and any of the immunized animals (either with rSsEno or rDPPIV) could be observed (*p* > 0.05).

### Evaluation of the antibody response

**Fig. 3:**
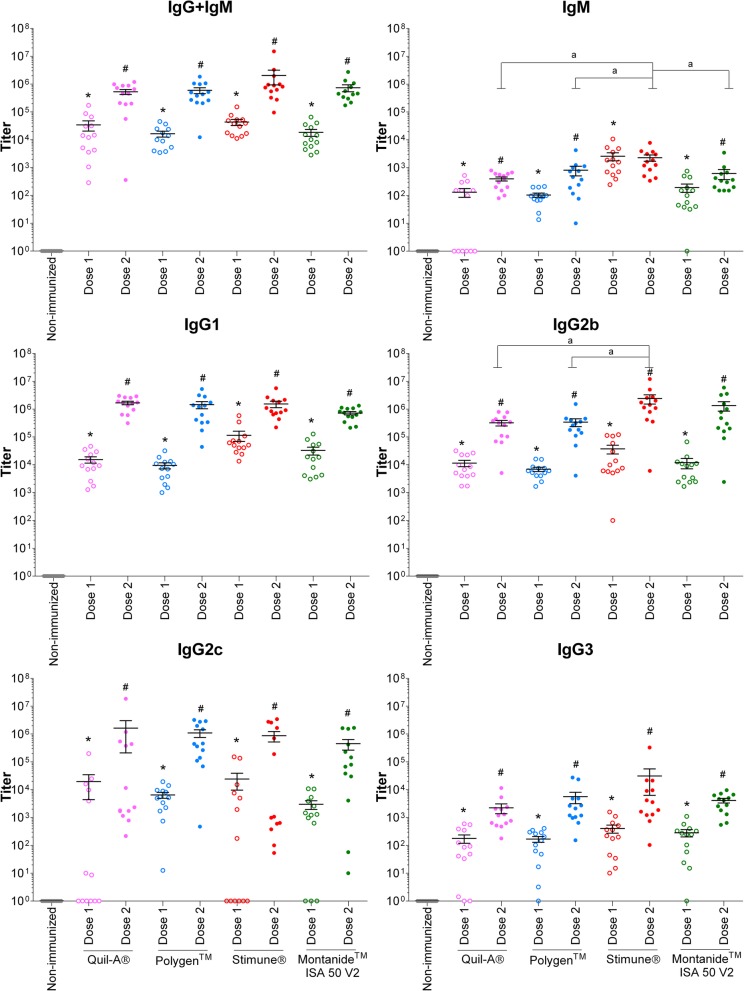
Titers of anti-Enolase antibody isotypes of mice immunized with rEnolase in combination with 4 adjuvants. Two 50 μg doses of rSsEno adjuvanted in Quil-A®, Polygen™, Stimune® or Montanide™ ISA 50 V2 were administered to mice. A control group (non-immunized) received 100 μL of phosphate-buffered saline. Isotypes were detected in sera twelve days after the first (Dose 1) and the second (Dose 2) vaccination. Data are expressed as mean ± standard error of the mean. * (*p* < 0.05) indicates a significant difference between non-immunized and dose 1. # (*p* < 0.05) indicates a significant difference between dose 1 and dose 2 and *a* (*p* < 0.05) between treated groups (adjuvants) for the second immunization (Dose 2)

**Fig. 4:**
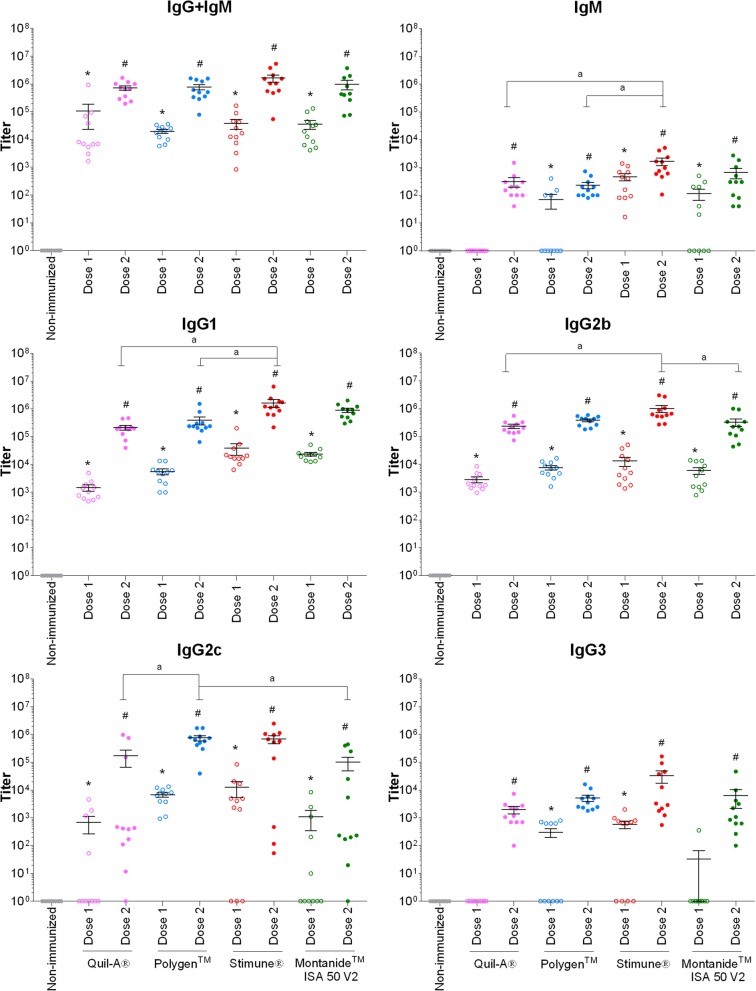
Titers of anti-DPPIV antibody isotypes of mice immunized with rDPPIV in combination with 4 adjuvants. Two 50 μg doses of rDPPIV adjuvanted in Quil-A®, Polygen™, Stimune® or Montanide™ ISA 50 V2 were administered to mice. A control group (non-immunized) received 100 μL of phosphate-buffered saline. Isotypes were detected in sera twelve days after the first (Dose 1) and the second (Dose 2) vaccination. Data are expressed as mean ± standard error of the mean. * (*p* < 0.05) indicates a significant difference between non-immunized and dose 1. # (*p* < 0.05) indicates a significant difference between dose 1 and dose 2 and *a* (*p* < 0.05) between treated groups (adjuvants) for the second immunization (Dose 2)

The efficacy of four different adjuvants in combination with either rSsEno or rDPPIV was compared regarding their capacity to induce the production of specific antibodies in a mouse model of infection. The production of specific antibodies against rSsEno and rDPPIV was evaluated before and after the first and second (boost) vaccination (Figs. 3 and 4). Overall, the four different adjuvants induced high total Ig titers (IgG + IgM) for both antigens after both vaccination doses (*p* < 0.05). Isotype switching (IgG1, IgG2b, IgG2c, and IgG3) was also observed for both antigens in the different combinations. However, the use of Stimune® gave a stronger antibody response against rSsEno after the first vaccination in the case of IgG1 or the booster for IgG2b in comparison to both Quil-A® and Polygen™ (*p* < 0.05), but not in comparison with Montanide™ ISA 50 V2. Stimune® was also able to induce a strong antibody response against rDPPIV after the first vaccination dose in the case of IgG3 compared to Quil-A® (p < 0.05) or the booster vaccination in the case of IgG1 and IgG2b in comparison with Quil-A® and Polygen™ or Quil-A® and Montanide™ ISA 50 V2, respectively (*p* < 0.05). Polygen™ enhanced IgG2c antibodies against rDPPIV after the first dose of the vaccine, compared to Quil-A® and Montanide™ ISA 50 V2. However, Montanide™ ISA 50 V2 induced a stronger IgG1 response against rDPPIV than Quil-A® for both vaccinations, as well as Polygen™ for the first vaccination only (*p* < 0.05). For both antigens, IgM production was lower than IgG production and a lighter booster effect was induced after the second vaccination, with the exception of Stimune® with rSsEno. However, Stimune® was, once again, the adjuvant giving one of the strongest IgM responses for both antigens (*p* < 0.05), even if there was no booster effect in combination with rSsEno. In general, and for both antigens, a relatively homogenous response was observed for IgM, IgG1 and IgG2b, but a higher heterogeneity was observed for IgG2c and IgG3. Finally, as expected, no detectable antibody titers against the proteins were observed in non-immunized mice (Figs. 3 and 4).

## Discussion

*S. suis* is considered one the most important swine bacterial pathogens in North America (25) and causes important economic losses to the swine industry worldwide (25). Control of *S. suis* disease is frustrating and complicated (10). Antibiotics can prevent clinical outbreaks, but the most effective belong to categories whose use in the swine industry has been greatly reduced given their importance in both human and veterinary medicine (26). In addition, the prophylactic and methaphylactic use of antibiotics in swine has been reduced or banished in several European countries. Control of stress factors, such as overcrowding, poor ventilation, high humidity, and inadequate sanitation, as well as the control of concomitant diseases (such as Porcine Reproductive and Respiratory Syndrome Virus), should be part of a global strategy to prevent clinical cases (25). However, in the presence of high virulent strains, protective vaccines are necessary.

The high heterogeneity of serotypes (and strains within a single serotype) has so far precluded the use of universal vaccines. Indeed, autogenous bacterins are mostly used in the field with questioned protection. Many studies in the last years have focused on the identification of cross-reacting proteins with protective capacities (10). Ideally, these proteins must be highly immunogenic and present in most field strains. In the present study, two proteins have been evaluated as such immunogens, whether the enolase and the DPPIV. In addition to its conserved functions in carbohydrate metabolism, the enolase has been suggested to be a virulence factor due to its capacity to bind to fibronectin, plasminogen and laminin, to enhance invasion of the blood-brain barrier and to induce pro-inflammatory cytokine production in the central nervous system (21, 22). In addition, the enolase has been shown to be protective for other pathogenic streptococci (27–29). In the present study, we showed that more than 85% of the strains tested reacted with a mono-specific antibody against rSsEno, which indicates that this protein is widely expressed by *S. suis* strains. Being an important proposed virulence factor and since it is widely present in field strains, the enolase would make an interesting protective candidate. However, results obtained so far are contradictory, showing either no or a relatively good protection by two different research groups (23, 24). In the present study, antibody production and protection in a mouse model of infection have been studied. Since it has been clearly proposed that the adjuvant used may influence the protection (and different IgG isotypes) obtained with a *S. suis* vaccine, we also tested four different adjuvants. Although small differences were observed, antibody response was generally high with all adjuvants, with a good response after the first dose and a booster response after the second vaccine dose. Whereas a strong IgG1 and IgG2b response was observed, a relatively lower and less homogenous response was observed for IgG2c and IgG3, confirming previous results obtained with other proteins (18). Independently of the antibody titer, no significant protection was observed with any of the adjuvants used. These results confirm those from Esgleas et al. (23), but are in contradiction with those obtained previously (24, 30). It is important to mention that the rSsEno was cloned from strain P1/7 strain in this study and that from Esgleas et al. (23), whereas strain 05ZYH33 was used in the successful protective studies of both Zhang et al. and Feng et al. (24, 30). There is 94% identity between P1/7 and 05ZYH33 enolase genes and in both cases, the complete gene was cloned into expression vectors. In the present study, the plasmid pET-32a used carries a C-terminal his-tag, while the plasmid pET28a used by Zhang et al carries two his-tags (C- and N-terminal) (24). In addition, an infection model of either inbreeding BALB/c (24, 30) or outbred CD-1 mice (Esgleas et al. (23) and the present study), were used. It is not possible to ascertain if such differences may be responsible for the discrepancy in the protective capacity of this recombinant protein. The use of inbred mice may influence the immune response of such animals to different pathogens (31). For example, BALB/c mice tend toward a Th2-predominant response (32). Indeed, outbred lines of mice are a better model to evaluate protection since they better represent the natural population (33).

The dipeptidyl aminopeptidase IV (DPPIV) is a serine protease that cleaves X-Pro/Ala di-peptide from the N-terminus of proteins and is present in *S. suis* (34). It has been recently demonstrated to bind fibronectin (35), although its role as a critical virulence factor is controversial (20, 35, 36). Interestingly, it has never been tested as a potential vaccine candidate. As was the case with the enolase, the DPPIV is largely present in *S. suis* field strains, which makes it an interesting vaccine candidate. However, results obtained in the present study are very similar to those obtained with the enolase: high antibody titers and good isotype switching, but lack of protection with the four adjuvants tested.

Finally, it should be considered that, although no protection with individual proteins were observed in the present study, it might be interesting to test if a combination of both sub-unit proteins with an appropriate adjuvant may induce some kind of protection. In addition, final confirmation using the natural host, the pig, would also be interesting. However, it should considered that so far, no single protein has been described as being non-protective in mice and protective in pigs.

## Conclusion

Although both proteins are present in most tested *S. suis* isolates, the recombinant proteins obtained in the present study did not confer protection with any of the adjuvant tested in an outbred model of infection despite the presence of specific IgM and different IgGs sub-types. These results confirm the difficulties in obtaining and evaluating (with good reproducibility) protective sub-unit protein candidates to control *S. suis* infections. Differences in the characteristics of the recombinant proteins produced, adjuvant used, animal model (animal species, inbred vs outbred), and *S. suis* strain used may significantly modify the obtained results. This problematic reinforces the concept and need of confirming published results by different research groups, as recently described for the role of critical virulence factors (37).

## Methods

### Bacterial strains and growth conditions

All strains and plasmids used in this study are listed in Table 1. The *S. suis* serotype 2, strain 31533, isolated from a pig with meningitis and previously used in protection studies, was chosen for challenging mice (38). The expression of SsENo and DPPIV proteins at the bacterial surface was evaluated by dot-blot. This strain was cultured as previously described with a few modifications (39). Briefly, bacteria were grown overnight onto sheep blood agar plates at 37 °C and isolated colonies were cultured in 5 mL of Todd–Hewitt broth (THB; Becton Dickinson, Mississauga, ON, Canada) for 8 h at 37 °C. Then, 10 μL of a 10^− 3^ dilution of 8 h-cultures were transferred into 30 mL of THB and incubated for 16 h at 37 °C. Stationary phase bacteria were washed in phosphate-buffered saline (PBS, pH 7.3). The bacterial pellet was then resuspended in THB and adjusted to the desired concentrations. *Escherichia coli* was grown in Luria-Bertani medium (Becton-Dickinson) containing 100 μg/mL of ampicillin (Sigma-Aldrich, Oakville, ON, Canada) when needed (38).

**Table 1:** Bacterial strains and plasmids used in this study

Strains/plasmids	General characteristics	Source/reference
*Escherichia coli*		
TOP10	F^−^ mrcA Δ(mrr-hsdRMS-mcrBC) φ80 lacZΔM15 ΔlacX74 recA1 araD139 Δ(ara-leu) 7697 galU galK rpsL (Str^R^) endA1 nupG	Invitrogen
BL21	F^−^ompT hsdS_B_ (r_B_^−^, m_B_^−^) gal dcm rne131 (DE3)	Invitrogen
*Streptococcus suis*		
31533	Virulent European serotype 2 ST1 strain isolated from pig with meningitis	(42)
Δ*dppIV*	Isogenic mutant strain derived from P1/7 strain. In frame deletion of *dppIV*	(20)
Group B *Streptococcus*		
COH-1	Capsular type III strain isolated from an infant with bacteremia	(40)
Plasmids		
pET-32a	Ap^r^, pBR322 *ori*, T7 promotor	Novagen
pBAD/thio	Ap^r^, pUC *ori*, P_BAD_ promotor	Invitrogen
pET-32aEnolase	pET-32a carrying *eno* gene for protein production	This work
pBAD/thioDPPIV	pBAD/thio-TOPO carrying dppIV gene for protein production	This work

A total of 359 *S. suis* field strains, belonging to different serotypes (Table 2), were tested for the expression of SsEno and DPPIV proteins as described below. These strains were isolated from the internal organs of diseased pigs in Canada and sent to the diagnostic laboratory of the Faculty of Veterinary Medicine of the University of Montreal. Bacteria were grown overnight onto sheep blood agar plates at 37 °C and isolated colonies were cultured under agitation in 5 mL of THB for 16 h at 37 °C at 120 rpm.

**Table 2:** Distribution of enolase and DPPIV among *S. suis* field strains

Serotype	Number of strains	Number of positive strains for Enolase	Number of positive strains for DPPIV
1	23	23	23
1/2	30	26	29
2	93	73	80
3	18	14	14
4	21	20	18
5	7	5	4
6	1	0	0
7	18	15	16
8	17	17	16
9	31	26	30
10	3	3	2
13	1	1	1
14	13	12	11
15	1	1	1
16	9	8	7
17	2	2	1
18	3	3	3
19	3	3	2
20	1	1	1
21	5	4	4
22	15	13	13
23	10	10	10
24	3	3	2
25	1	1	1
27	3	3	3
28	7	6	7
29	4	3	4
30	4	4	4
31	3	2	2
32	1	1	0
33	4	4	2
34	4	3	4

### Cloning, expression and purification of rSsEno and rDPPIV proteins

The cloning and purification of the protein candidates were performed based on the methodology previously described (21, 34). Briefly, the sequence used to design primers for PCR amplification of the SsEno gene was the SsEno1-forward primer 5′-TATAA**GGATCC**TATAAGGATCCTTGTCAATTATTACTGATGTTTACGC-3′, introducing a BamHI site (bold and underlined letters), and the Eno2-reverse primer 5′-TATA**AAGCTT**TTATTTTTTCAAGTTGTAGAATGAGTTCAAGCC-3′, introducing a HindIII site (bold and underlined letters). The PCR amplified gene was confirmed by automated sequencing and cloned into pET-32a vector (Novagen, Madison, WI, USA), using the BamHI and HindIII sites. The plasmid pET-32a*SsEno* was transformed into *E. coli* Bl21DE3 for IPTG-inducible expression of recombinant SsEno. The protein was purified by affinity chromatography using the His-Bind Resin Chromatography Kit (Novagen, Madison, WI, USA) according to manufacturer’s instructions. In the case of the DPPIV, the selected gene was amplified by PCR using the following primers: 5′ CGCTTTAATCAATTTTCTTTCATAAAAAAAGAGAC 3′ and 5′ TTTGGATTTTCATTGAGTATTAGTGCG 3′ and stop codons in concordance with the pBAD/thio-TOPO system (Invitrogen, Carlsbad, CA, USA). Cytosol extract were then purified in two steps, whether a Mono-Q 5/50 GL (GE Healthcare, Baie d’Urfé, Quebec, Canada) followed by a concentration with Superdex 200 10/300 GL (GE Healthcare), according to manufacturer’s instructions. Previous data clearly showed that this technique lead to higher yields than using a Ni^2+^-nitrilotriacetic acid affinity chromatography column.

For both vaccine candidates, protein-containing fractions were determined by SDS-PAGE and Western blot using an anti-His-tag antibody (R&D Systems, Minneapolis, MN, USA) and were dialysed. The purified recombinant proteins were then concentrated using Amicon Ultra-15 (Millipore, Billerica, MA, USA) and protein quantification was evaluated using the Pierce Bicinchoninic Acid Protein Assay Kit (Fisher Scientific, Ottawa, ON, Canada). Two mono-specific polyclonal hyperimmune sera were then produced in rabbits using the purified rSsEno and rDPPIV protein, respectively, and tested by immunoblot using the purified proteins as previously described (19).

### Detection of naturally expressed SsENo and DPPIV proteins on the surface of *S. suis* field strains

Centrifugation of 5 mL *S. suis* overnight cultures in THB was performed and bacterial pellets were resuspended in PBS before treatment with 0.5% formaldehyde for 1 h at 37 °C. Ten μl of a formalin-killed whole bacteria suspension were blotted on a Nitrocellulose Western blotting membrane (Bio-Rad, Hercules, CA, USA). The membrane was blocked for 1 h with a solution of Tris-buffered saline (TBS) containing 2% casein, followed by 2 h incubation with mono-specific polyclonal hyperimmune rabbit serum against either SsEno or DPPIV, diluted in blocking buffer. The membrane was then washed with TBS and further incubated for 1 h with a goat anti-rabbit IgG horseradish peroxidase (HRP)-conjugated antibody (Jackson ImmunoResearch, West Grove, PA). The membrane was rinsed with TBS and revealed using a 4-chloro-1-naphthol solution (Sigma-Aldrich). The *S. suis* 31533 strain was used as a positive control. Group B *Streptococcus* serotype III COH-1 strain (40) and a *S. suis* isogenic mutant defective in the production of DPPIV (20) were used as negative controls for SsEno and DPPIV detection, respectively.

### Immunization and protection studies

All experiments involving mice were conducted in accordance with the guidelines and policies of the Canadian Council on Animal Care and the principles set forth in the *Guide for the Care and Use of Laboratory Animals* by the Animal Welfare Committee of the University of Montreal (RECH-1748). Housing and husbandry of animals were taken care by the personal of level II facilities at the University of Montreal. Six-week-old CD-1 mice (Charles River Laboratories, Saint-Constant, QC, Canada) were randomly assigned to five groups of 11 or 13 mice for immunization with rSsEno or rDPPIV, respectively, as accepted by the ethical committee. Animals were immunized twice subcutaneously at a 2-week interval with either 50 μg of rSsEno or rDPPIV mixed with one of the following adjuvants: 20 μg of Quil-A® (Brenntag Biosector, Frederikssund, Danemark), 15% of Polygen™ (MVP Laboratories, Omaha, NE, USA), 55% of Stimune® (Thermo Fisher Scientific, Waltham, MA, USA) or 50% of Montanide™ ISA 50 V2 (Seppic, Paris, France), following the manufacturers’ recommendations. A control group received 100 μL of PBS. To follow antibody responses, mice were bled (100 μL) before immunization, and twelve days after the first and the second vaccination doses by the dorsal tail vein. Fifteen days after the second vaccination, animals were intraperitoneally challenged as described below.

### Bacterial challenge

A mouse model of infection was used (38, 39). These studies were carried out in strict accordance with the recommendations of and approved by the University of Montreal Animal Welfare Committee guidelines and policies, including euthanasia to minimize animal suffering through the use of humane endpoints, applied throughout this study when animals were seriously affected since mortality was not an endpoint measurement. Immunized and control mice were inoculated with 4 × 10^7^ CFU via the intraperitoneal route. Health and behavior were monitored at least thrice daily until 72 h post-infection and twice thereafter until the end of the experiment (14 days post-infection) for the development of clinical signs of sepsis, such as depression, swollen eyes, rough hair coat, prostration, and lethargy. Mice were immediately euthanized upon reaching endpoint criteria using CO2 followed by cervical dislocation. No mice died before meeting endpoint criteria and all surviving mice were euthanized as described above at the end of the experiment (14 days p.i.).

### Antibody titration

Polysorp immunoplates (Nunc, Roskilde, Denmark) were coated with a solution of 0.3 μg/mL rSsEno ou rDPPIV in carbonate buffer (0.1 M [pH 9.6] at 100 μL/well) for 1 h at 37 °C. After washing with PBS containing 0.05% Tween 20 (PBS-T), mouse sera were serially diluted (2-fold) in PBS-T and incubated for 90 min at room temperature (RT). Plates were then washed in PBS-T and incubated with peroxidase-conjugated goat anti-mouse total Ig (IgG plus IgM) (Jackson ImmunoResearch), IgM, IgG1, IgG2b, IgG2c or IgG3 (Southern Biotech, Birmingham, AL, USA) antibodies for 1 h at RT. Isotype IgG2a was not measured as it is considered to be homologous to IgG2c in mice (41). After washing, plates were developed with 3,3′,5,5′-tetramethylbenzidine (TMB) substrate and the enzyme reaction was stopped by addition of 1 M H_2_SO_4_. Absorbance was read at 450 nm with an ELISA plate reader. The reciprocal of the last serum dilution that resulted in an optical density at 450 nm (OD_450_) of ≤0.1 (cutoff) was considered the titer of that serum.

### Statistics

Data are expressed as mean ± standard error of the mean (SEM). Significant differences in between groups were determined using the t-test and one-way ANOVA, where appropriate. For in vivo virulence experiments, survival was analyzed using the LogRank test. A *p* < 0.05 was considered statistically significant.

## Data Availability

The data and materials not presented in this manuscript are available from the corresponding author upon request.

## Abbreviations

CPS: capsular polysaccharide
DPPIV: dipeptidyl peptidase IV
HRP: horseradish peroxidase
PBS: phosphate-buffered saline
rDPPIV: recombinant dipeptidyl peptidase IV
rSsEno: recombinant enolase
SEM: Standard error of the mean
SsEno: enolase
TBS: Tris-buffered saline
THB: Todd-Hewitt broth
TMB: 3,3′,5,5′-tetramethylbenzidine
